# Effects of drought on the microtranscriptome of field-grown sugarcane plants

**DOI:** 10.1007/s00425-012-1795-7

**Published:** 2012-11-06

**Authors:** Agustina Gentile, Thaís H. Ferreira, Raphael S. Mattos, Lara I. Dias, Andrea A. Hoshino, Monalisa S. Carneiro, Glaucia M. Souza, Tercílio Calsa, Rejane M. Nogueira, Laurício Endres, Marcelo Menossi

**Affiliations:** 1 Laboratório de Genoma Funcional, Departamento de Genética, Evolução e Bioagentes, Instituto de Biologia, Universidade Estadual de Campinas, Campinas, SP Brazil; 2 Departamento de Biotecnologia Vegetal, Centro de Ciências Agrárias, Universidade Federal de São Carlos, Araras, SP Brazil; 3 Departamento de Bioquímica, Instituto de Química, Universidade de São Paulo, São Paulo, Brazil; 4 Laboratório de Genômica e Proteômica de Plantas, Departamento de Genética, Centro de Ciências Biológicas, Universidade Federal de Pernambuco, Recife, PE Brazil; 5 Laboratório de Fisiologia Vegetal, Departamento de Biologia, Universidade Federal Rural de Pernambuco, Recife, PE Brazil; 6 Centro de Ciências Agrárias, Universidade Federal de Alagoas, Rio Largo, AL Brazil

**Keywords:** MicroRNAs, Bioenergy, Sugarcane, Drought stress, Field conditions, Solexa sequencing

## Abstract

Sugarcane (*Saccharum* spp.) is the most promising crop for renewable energy. Among the diverse stresses that affect plant productivity, drought stress frequently causes losses in sugarcane fields. Although several studies have addressed plant responses to drought using controlled environments, plant responses under field conditions are largely unknown. Recently, microRNA (miRNA)-mediated post-transcriptional regulation has been described as an important and decisive component in vegetal development and stress resistance modulation. The role of miRNAs in sugarcane responses to drought under field conditions is currently not known. Two sugarcane cultivars differing in drought tolerance were grown in the field with and without irrigation (rainfed) for 7 months. By using small RNA deep sequencing, we were able to identify 18 miRNA families comprising 30 mature miRNA sequences. Among these families, we found 13 mature miRNAs that were differentially expressed in drought-stressed plants. Seven miRNAs were differentially expressed in both cultivars. The target genes for many of the differentially expressed mature miRNAs were predicted, and some of them were validated by quantitative reverse transcription PCR. Among the targets, we found transcription factors, transporters, proteins associated with senescence, and proteins involved with flower development. All of these data increase our understanding of the role of miRNAs in the complex regulation of drought stress in field-grown sugarcane, providing valuable tools to develop new sugarcane cultivars tolerant to drought stress.

**Electronic supplementary material:**

The online version of this article (doi:10.1007/s00425-012-1795-7) contains supplementary material, which is available to authorized users.

## Introduction

Sugarcane (*Saccharum* spp.) is a source of sugar and ethanol. This biofuel has been increasingly acknowledged as the most promising energy substitute for oil. Apart from being very productive, sugarcane is largely affected by biotic and abiotic stresses that lead to decreased yields (Boyer 1982; Maybank et al. 1995). Drought stress is one of the most important stresses that affect crops in many areas in the world. Under severe conditions, drought can produce irreversible alterations that could induce plant death. Drought causes several changes in sugarcane, such as the inhibition of root development, reduction in water and nutrient uptake, the decrease of leaf and stalk elongation, and in some cultivars, leaf rolling, which interferes with light absorption, reducing photosynthesis (Inman-Bamber and Smith 2005).

MicroRNAs (miRNAs) are an extensive family of small RNAs with a unique biogenesis (Axtell 2008; Voinnet 2009). They are small (19–24 nucleotides, nt), endogenous, single-stranded non-coding RNA molecules that hybridize to target mRNA and direct site-specific cleavage or translational repression (Carrington and Ambros 2003; Kidner and Timmermans 2006). MicroRNAs have been described as regulatory non-coding RNAs in plants and animals (Bartel 2004; Carrington and Ambros 2003). In plants such as *Arabidopsis*, *Brachypodium*, rice and maize, several studies have described miRNA genes as well as their targets in a wide variety of tissues, developmental stages, and treatment conditions (Gustafson et al. 2005; Jones-Rhoades and Bartel 2004; Rhoades et al. 2002; Unver and Budak 2009; Wu et al. 2009; Zhang et al. 2009a, b).

Because of their ability to regulate gene expression, many studies have focused on miRNAs. Plant miRNAs are often identical across large evolutionary distances (Axtell and Bartel 2005; Floyd and Bowman 2004) and are highly complementary to their targets, and this complementarity can be used to identify them using bioinformatics approaches (Fahlgren and Carrington 2010; Reinhart et al. 2002; Rhoades et al. 2002). Many predicted miRNA target genes encode regulatory proteins, suggesting that they function as important regulators (Bartel 2004). In *Arabidopsis*, 68 % of the predicted conserved targets encode transcription factors that appeared to be involved in developmental patterning or stem cell identity (Jones-Rhoades et al. 2006). The same was reported in *Brachypodium distachyon*, where the majority of the predicted target genes encode transcription factors regulating plant development, morphology and flowering time (Unver and Budak 2009). Some reports revealed miRNA involvement in gene regulation under drought stress in rice (Zhao et al. 2007b) and maize (Zhang et al. 2009b). Because of the differences between animal and plant miRNAs in biogenesis, targets and mode of repression, it has been suggested that they originated independently in each kingdom (Axtell 2008). However, recent observations in the unicellular green algae *Chlamydomonas reinhardtii* support a more complex evolution (Molnar et al. 2007; Zhao et al. 2007c).

MicroRNA expression studies were greatly facilitated by the improvement of large-scale sequencing technologies that have been used in many studies (Pantaleo et al. 2010; Ruby et al. 2006; Wei et al. 2009; Zhang et al. 2009a; Zhao et al. 2010). The Solexa technique allows for low-cost, high-quality and robust parallel sequencing of millions of 36 base-long fragments (Bentley 2006; Shendure et al. 2005). This methodology allows us to assess the expression profile of miRNAs by digital gene expression tag profiling (DGE). It is assumed that the number of times a particular sequence is observed in a cDNA sequencing library indicates the amount of that transcript in the sample. Through basic statistical tests, it is possible to compare the expression profiles of two samples. Recently, Hoen et al. (2008) obtained evidence that DGE detects more expression differences with fewer false-positives than quantitative real-time PCR and microarrays. To date, the majority of known plant miRNA sequences belong to *Arabidopsis thaliana*, *Oryza sativa*, and *Populus trichocarpa* because of their sequenced genomes. In this study, two field-grown sugarcane cultivars showing different responses to drought stress were analyzed. To the best of our knowledge, this is the first study involving sugarcane plants grown under field conditions and submitted to drought stress. The differentially expressed miRNAs were identified by high-throughput sequencing, and the miRNA targets were predicted in silico. Some of the targets were validated by quantitative reverse transcription PCR (RT-qPCR).

## Materials and methods

### Plant samples

Sugarcane cultivars RB867515 (high tolerance to drought, HT) and RB855536 (lower drought tolerance, LT) from RIDESA (Rede Interuniversitária para o Desenvolvimento do Setor Sucroenergetico) were field-grown in Campo Alegre, Alagoas, Brazil (9°45′32″ S, 36°13′09″ W), and samples were collected on 04 April 2009, 7 months under irrigation or without irrigation (rainfed). The cultivars RB867515 (RB72454 × *****?) and RB855536 (SP70-1143 × RB72454) are derived from half-sib families and exhibit different responses to water deficit. The root system of the LT cultivar (RB855536) is less developed in deeper soil layers (20–80 cm) (Santos 2010; Vasconcelos et al. 2003). The length/root mass ratio is one of the RB855536 phenotypic features that indicates the lower tolerance to drought. The roots from the cultivar HT (RB867515) shows a homogeneous distribution throughout the layers of the soil profile (0–80 cm), favoring a higher tolerance to drought (Santos 2010). Sugarcane plants were grown at field conditions during the dry season (Supplementary Fig. S1). Irrigated plots received 60 mm of irrigation every month. In irrigated plants, samples were collected 5 days after applying water to the fields, ensuring that plants were well watered. Rainfed plants experienced water deficit throughout the 7-month period, except in February 2009, when plant rainfall matched plant water demands (Supplementary Fig. S1). At the time point plant samples were collected, plants were experiencing a water deficit (Supplementary Fig. S1) and the last rain occurred 15 days before (data not shown). Several physiological parameters evidenced that rainfed plants were under drought stress (Supplementary Fig. S1). Leaf + 1 tissue (the highest expanded leaf with a visible dewlap) was collected in quadruplicate after 7 months from irrigated and rainfed drought-stressed plants. Samples were snap-frozen and maintained at −80 °C. Two replicates were combined and used for Solexa sequencing.

### Leaf total RNA extraction

Total RNA was isolated using the miRVana™ miRNA isolation kit (Life Technologies, USA) according to manufacturer’s protocol, with minor modifications. Briefly, ten volumes of lysis/binding buffer per macerated leaf-tissue mass were added into a tube and mixed. One volume of miRNA homogenate was added to the tissue lysate and mixed by vortexing. After 10 min on ice, ten volumes of acid-phenol:chloroform were added and mixed gently. The samples were then centrifuged for 7 min at 10,000×*g* at room temperature to separate the aqueous and organic phases. The aqueous phase was removed carefully and transferred to a new tube. A 1.25× volume of absolute ethanol was added to the aqueous phase, mixed, and placed onto the filter cartridge. Samples were centrifuged 20 s at 10,000×*g* to pass the mixture through the filter. The samples on the filters were then washed, the filter transferred to a new tube and the RNA eluted in 80 μL of pre-heated nuclease-free water. Total RNA samples were quantified (NanoDrop, Thermo Scientific, USA) and stored at −80 °C for later use.

### Small RNA sequencing

The cDNA library synthesis and sequencing were performed at BGI (Beijing Genomic Institute, Tai Po, Hong Kong) using the Solexa platform. Briefly, total RNA samples received at the company were analyzed in a 2100 Bioanalyzer (Agilent, USA) to check for integrity and quality. Before constructing the miRNA libraries, RNAs from 16 to 27 nucleotides long were selected by polyacrylamide gel electrophoresis, ligated with adaptors at both ends and the products used for cDNA synthesis. Then, they were PCR-amplified and sequenced using the Solexa technology.

### Bioinformatics analysis

The prediction of the sugarcane precursors (pre-miRNAs) was performed by searching for sequences that matched with the validated mature miRNAs. The adaptors were then removed, and any reads shorter than 19 nucleotides or longer than 24 nucleotides were discarded. The raw data with all sequences used in this work may be available upon request. The transcripts were mapped to the *Sorghum bicolor* genome and sugarcane transcriptome as references using the miRDeep-P program (Yang and Li 2011). For a given mapped read, the optimal window size was 250 bp, which was used to extract reference sequences for predicting the RNA secondary structure (Yang et al. 2011). The miRDeep core algorithm with a plant-specific scoring system based on the known characteristics of plant miRNA genes was used to find the secondary structures of the sequences (Meyers et al. 2008). RNA sequences were considered miRNA precursor candidates if the following conditions were met: the RNA sequence could fold into the characteristic stem-loop hairpin secondary structure, the mature miRNA lays within one arm of the hairpin structure and had a maximum of six mismatches with the miRNA* sequence in the opposite arm, the predicted secondary structures had negative MFEs, and the G/C content was between 30 and 70 % (Zanca et al. 2010). All the secondary structures of the precursors were predicted using the RNAfold program (Hofacker 2003).

After the normalization of the number of reads, the expression of each miRNA was calculated based on the Audic–Claverie method (Audic and Claverie 1997). The target of each miRNA was predicted by psRNATarget (http://plantgrn.noble.org/psRNATarget/), which searches for target genes based on complementarity scoring and secondary structure analysis (Dai and Zhao 2011).

### Evaluation of miRNA and miRNA targets expression profiles by RT-qPCR

RT-loop primers (loop-RT), forward specific PCR primers (loop-FW) and reverse universal primers were designed following Chen et al. (2005) (Supplementary Table S1) for reverse transcription and PCR amplification of sugarcane miRNAs, of two sugarcane genes related to drought stress, encoding DREB and dehydrin homologs, and to validate some of the target genes. Reverse transcriptase reactions were performed as described by Varkonyi-Gasic et al. (2007). Each reaction contained 2.5 μg of DNA-free total RNA, 1 μL of each RT-loop primer (1 μM), 1 μL oligo d(T)_17_VN (50 μM), and 1 μL of dNTP mix (10 μM). The reaction was incubated for 10 min at 65 °C and then placed on ice for 2 min. Subsequently, 5X First Strand Buffer, DTT, RNAseOut, and Superscript III enzyme (Life Technologies, USA) were added. This reaction was incubated in a Verity^TM^ Thermal Cycler (Applied Biosystems, USA) for 30 min at 16 °C, followed by 60 cycles of 30 °C for 30 s, 42 °C for 30 s, and 50 °C for 1 s. Finally, the reaction was incubated 5 min at 85 °C for the enzyme inactivation.

Real-time PCR was performed to analyze the expression of sugarcane genes. The reactions were carried out using the SYBR Green PCR Master Mix (Applied Biosystems, USA) on 7500 Real-Time PCR System (Applied Biosystems, USA). Each 18 μL PCR reaction included 2 μL of cDNA, 10 μL of SYBR Green Master Mix (1×), 1 μL of forward primer (10 μM), 1 μL of reverse primer (10 μM) (Supplementary Table S1), and water. The polyubiquitin gene (Papini-Terzi et al. 2005) was used as a reference. The reactions were performed at 95 °C for 10 min, followed by 40 cycles of 95 °C for 15 s and 60 °C for 1 min with a final dissociation curve analysis. All reactions were run in triplicate with three biological replicates.

The real-time PCR data analysis was performed based on the reaction efficiencies required to calculate the fold-changes and using the web-based QPCR system (Pabinger et al. 2009).

## Results

### Physiological data and confirmation of drought stress in sugarcane plants

Plants from the cultivars RB867515 (HT to drought) and RB855536 (LT to drought) were grown in the field for 7 months with and without irrigation (rainfed). Water deficit negatively affected the photosynthetic activity and reduced the stomatal conductance (gs), photosynthesis (A), transpiration rate (E), and water and osmotic potentials (Ψ_w_ and Ψ_o_), indicating a reduction in the photosynthetic performance (Supplementary Fig. S1). For all of the analyzed parameters, the HT cultivar was less affected by drought stress, demonstrating a better response under adverse conditions (Supplementary Fig. S1).

**Fig. 1:**
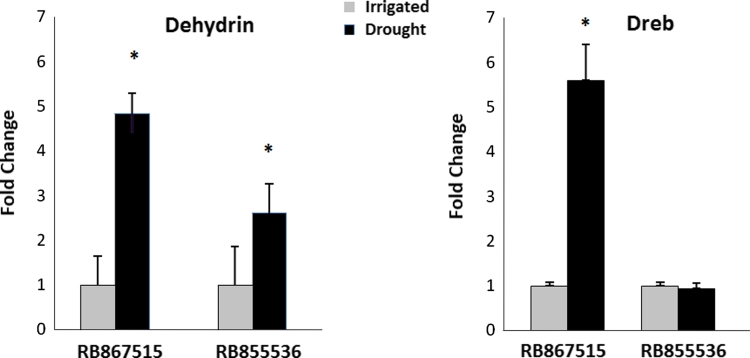
RT-qPCR of two sugarcane genes encoding dehydrin (SCQGLR1085F11.g) and the DREB transcription factor (SAS: SCJLLR2013H07.g). RB867515 (higher drought tolerance, HT) and RB855536 (lower drought tolerance, LT) plants were irrigated (*grey bars*) or subjected to water deficiency by withholding irrigation (*black bars*) for 7 months. RB867515-irrigated was used as reference sample to calculate the fold change. *Error bars* represent the standard error (*n* = 2), * *p* < 0.05. Statistics were calculated between irrigated and non-irrigated treatments in each cultivar using a permutation test. The expression in irrigated RB867515 plants was considered as 1

To further confirm that plants were stressed, we evaluated the expression of a sugarcane gene encoding a dehydrin (Sugarcane Assembled Sequence, SAS: SCQGLR1085F11.g) by RT-qPCR (Fig. 1). This gene was already described as drought-induced in sugarcane (Rocha et al. 2007). A second gene encoding a homolog of a DREB transcription factor (SAS: SCJLLR2013H07.g) was also used. This class of transcription factor was reported to be induced by cold and dehydration in plants (Agarwal et al. 2006). In sugarcane plants, the dehydrin gene was induced in both cultivars and showed higher levels in the HT cultivar RB867515 (Fig. 1). Interestingly, the DREB homolog was upregulated only in the HT cultivar (Fig. 1). As expected, these data show that drought was affecting the sugarcane transcriptome.

### Identification of sugarcane miRNAs under water deficit

**Fig. 2:**
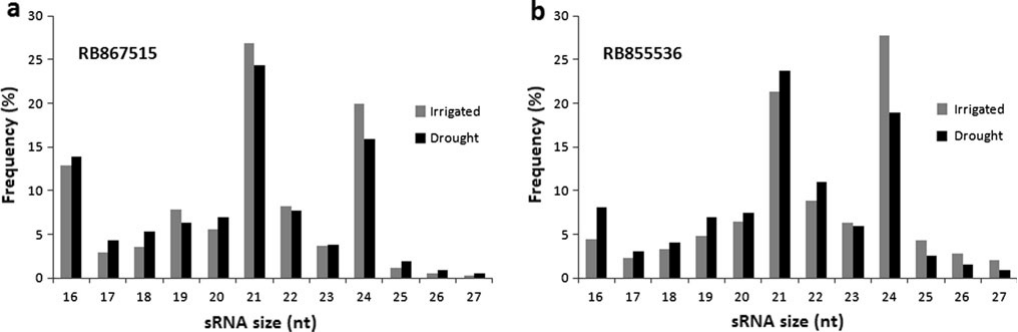
Size distribution of small RNA (sRNA) sequences in sugarcane. Plants of two cultivars, RB867515 (**a**) and RB855536 (**b**), were field-grown for 7 months in two conditions irrigated and drought-stressed. RB867515 is known as higher drought tolerant (HT) and RB855536 as lower drought tolerant (LT). The sRNA size is shown in number of nucleotides (nt)

To identify miRNAs from *Saccharum* spp., a high-throughput approach using Solexa sequencing was used, and the sequences were compared to the miRBase database. To this end, total RNA samples were obtained from the mature leaves of two different sugarcane cultivars, RB867515 (HT) and RB855536 (LT), that were grown in the field for 7 months with (I) or without (D) irrigation. These four cDNA libraries yielded between 8 and 12 million clean reads each (Table 1) and more than 40 million reads in total. Good-quality reads between 18 and 25 bases were analyzed. In both cultivars under either condition (irrigated and stressed), the most abundant sRNAs were either 21-nt or 24-nt long, representing miRNAs and siRNAs, respectively (Fig. 2). This pattern was also observed in sequencing analyses from other plant species (Chapman and Carrington 2007). After annotation of the unique tags using the RFam and GenBank RNA databases, the remaining tags were compared to sorghum miRNAs in miRBase, which resulted in 18 families and 30 mature miRNA sequences (Table 2).

**Table 1:** Small RNA deep-sequencing results for sugarcane leaves from RB867515 (HT) and RB855536 (LT) cultivars under irrigation (I) and drought (D) conditions after 7 months of stress on the field

Category	HTI				HTD			
	Unique RNAs	Percent (%)	Total RNAs	Percent (%)	Unique RNAs	Percent (%)	Total RNAs	Percent (%)
miRNA	33,295	0.86	2,568,341	21.04	15,074	0.71	1,547,690	20.47
rRNA	57,826	1.49	690,977	5.66	80,838	3.8	1,307,606	17.29
siRNA	99,469	2.57	640,673	5.24	31,807	1.49	208,823	2.76
snRNA	2,778	0.07	13,047	0 1	2,893	0.14	14,372	0.19
snoRNA	1,457	0.04	4,727	0.04	1,05	0.05	3,2938	0.04
tRNA	15,783	0.41	1,058,986	8.67	17,416	0.82	479,527	6.34
Unannotated	3,660,105	94.55	7,230,626	59.23	1,978,922	92.99	4,000,025	52.9
Total small RNAs	3,870,713	100.00	12,207,377	100.00	2,128,000	100.00	7,561,341	100.00

Category	LTI				LTD			
	Unique RNAs	Percent (%)	Total RNAs	Percent (%)	Unique RNAs	Percent (%)	Total RNAs	Percent (%)
miRNA	15,663	0.52	1,294,640	12.98	19,966	0.72	1,853,916	17.76
rRNA	108,411	3.57	1,994,571	20.00	83,009	2.99	1,604,192	14.41
siRNA	55,071	1.81	402,188	4.03	44,402	1.6	255,345	2.45
snRNA	4,955	0.16	43,655	0.44	3,797	0.14	21 34	0.21
snoRNA	2,184	0.07	10,697	0.11	2,624	0.09	18,403	0.18
tRNA	23,831	0.78	766,089	7.68	19,327	0.7	547,433	5.25
Unannotated	2,825,979	93.08	5,461,047	54.76	2,605,783	93.77	6,235,247	59.75
Total small RNAs	3.036.094	100.00	9,972,887	100.00	2,778,908	100.00	10,436,376	100.00

**Table 2:** Sugarcane miRNA families and mature miRNAs identified by Solexa sequencing

miR family	miRNA name	Mature sequences	Sorghum precursor
miR156	ssp-miR156-seq 1	UUGACAGAAGAGAGUGAGCAC	sbi-MIR156a
			sbi-MIR156b
			sbi-MIR156c
	ssp-miR156-seq 2	UGACAGAAGAGAGCGAGCAC	sbi-MIR156e
	ssp-miR160-seq 1	UGCCUGGCUCCCUGUAUGCCA	sbi-MIR160c
			sbi-MIR160a
			sbi-MIR160d
miR160	ssp-miR160-seq 2	UGCCUGGCUCCCUGAAUGCCA	sbi-MIR160f
	ssp-miR160-seq 3	AGGUAGAGGAGAAGAGUG	sbi-MIR160b
miRl64	ssp-miR164	UGGAGAAGCAGGGCACGUGCU	sbi-MIR164b
miR166	ssp-miR166-seq 1	UCGGACCAGGCUUCAUUCCCC	sbi-MIR166b
			sbi-MIR166c
			sbi-MIR166d
	ssp-miR166-seq *2*	UCGGACCAGGCUUCAUUCCUC	sbi-MIR166f
			sbi-MIR166k
miR167	ssp-miR166-seq 3	UCGGACCAGGCUUCAAUCCCU	sbi-MIR166e
			sbi-MIR166f
			sbi-MIR166g
	ssp-miR167b	UGAAGCUGCCAGCAUGAUCU	sbi-MIR167a
		UGAAGCUGCCAGCAUGAUCUGA	sbi-MIR167g
			sbi-MIR167h
		UGAAGCUGCCAGCAUGAUCUG	sbi-MIR167c
miR168	ssp-miR168a	UCGCUUGGUGCAGAUCGGGAC	sbi-MIR168
miR169	ssp-miR169-seq 1	GGGCAAAUCAUCCGGGCUAGC	sbi-MIR169o
	ssp-miR169-seq 2	CGGCAAGUUGUUCUUGGCUAC	sbi-MIR169a
miR171	ssp-miR171-seq 1	UUGAGCCGCGUCAAUAUCUCC	sbi-MIR171h
	ssp-miR171-seq *2*	UGAUUGAGCCGUGCCAAUAUC	sbi-MIR171i
miR172	ssp-miR172	AGAAUCUUGAUGAUGCUGCAU	sbi-MIR172d
miR393	ssp-miR393	CUCCAAAGGGAUCGCAUUGAU	sbi-MIR393b
miR394	ssp-miR394	UUGGCAUUCUGUCCACCUCC	sbi-MIR394b
miR395	spp-miR395-seq 1	GUUCCCUGCAAGCACUUCACA	sbi-MIR395b
			sbi-MIR395a
			sbi-MIR395c
			sbi-MIR395e
			sbi-MIR395f
			sbi-MIR395g
			sbi-MIR395h
			sbi-MIR395i
			sbi-MIR395d
	spp-miR395-seq 2	UGAAGUGUUUGGGGGAACUC	sbi-MIR395i
			sbi-MIR395j
			sbi-MIR395k
miR396	ssp-miR396	UUCCACAGCUUUCUUGAA	sbi-MIR396b
miR397	ssp-miR397	UUGACUGCAGCGUUGAUGAGC	sbi-MIR397
miR399	ssp-miR399-seq 1	UGCCAAAGGAGAGUUGCCCU	sbi-MIR399i
	ssp-miR399-seq 2	UGCCAAAGGAGAAUUGCCC	sbi-MIR399a
			sbi-MIR399h
			sbi-MIR399j
	ssp-miR399-seq 3	GUGCAGCUCUCCUCUGGCAUG	sbi-MIR399b
miR528	ssp-miR528	UGGAAGGGGCAUGCAGAGGAG	sbi-MIR528
miR529	ssp-miR529	AGAAGAGAGAGAGUACAGCCU	sbi-MIR529
miR1432	ssp-miR1432	UCAGGAAAGAUGACACCAA	sbi-MIR1432

miRNAs were found in the leaves of two sugarcane cultivars, one with higher tolerance to drought (HT, RB867515) and the other with lower tolerance to drought (LT, RB855536). Two mismatches were allowed using sorghum mature miRNAs as references

### Bioinformatics identification of sugarcane miRNA precursors

The whole set of miRNA sequences shown in Table 2 was mapped onto SoGI and SUCEST databases to identify their precursors. In total, eight precursors corresponding to seven miRNA families were found, and these corresponded to precursors already deposited in miRBase (Ferreira et al. 2012; Zanca et al. 2010). Seven precursor sequences were from the SUCEST (http://sucest-fun.org/) database, whereas only one was found in the SoGI (http://compbio.dfci.harvard.edu/) database (data not shown). Two additional miRNA precursors (ssp-MIR168 and ssp-MIR396) were found in comparison with our previous work with sugarcane plants grown in glasshouses (Ferreira et al. 2012). All of the precursor sequences found in both databases have the capacity to fold into hairpin structures and hold the mature miRNA in one arm of the hairpin structure, which, together with the negative MFEs energy values and the G/C content, supports the veracity of the sugarcane precursors (Ferreira et al. 2012; Zanca et al. 2010).

### Differential expression of sugarcane miRNAs under drought stress

We have identified 13 differentially expressed mature miRNAs, using a *p* value <0.05 and fold change ≥2 (Table 3). The HT cultivar had 11 miRNAs that were differentially expressed between the irrigated and drought-stressed plants (HTI × HTD), while the LT cultivar had nine miRNAs modulated by drought stress (LTI × LTD). Among the 11 miRNAs found in the HT cultivar, 3 were upregulated (ssp-miR160-seq 3, ssp-miR399-seq 3, and ssp-miR528), and 8 were downregulated (ssp-miR166-seq 3, ssp-miR169-seq 2, ssp-miR171-seq 2, ssp-miR172, ssp-miR393, ssp-miR396, ssp-miR399-seq 2, and ssp-miR1432). In the LT cultivar, six were upregulated (ssp-miR160-seq 1, ssp-miR160-seq 3, ssp-miR394, ssp-miR399-seq 2, ssp-miR399-seq 3, and ssp-miR1432), and three were downregulated (ssp-miR166-seq 3, ssp-miR171-seq 2, and ssp-miR396). Both cultivars shared seven differentially expressed miRNAs in this experiment: ssp-miR160-seq 3, spp-miR166-seq 3, ssp-miR171-seq 2, ssp-miR396, ssp-miR399-seq 2, spp-miR399-seq 3, and ssp-miR1432. Among them, five were induced or repressed in both cultivars (ssp-miR160-seq 3, spp-miR166-seq 3, ssp-miR171-seq 2, ssp-miR396, and spp-miR399-seq 3), while the other two (ssp-miR399-seq 2 and ssp-miR1432) had the opposite patterns (Table 3).

**Table 3:**
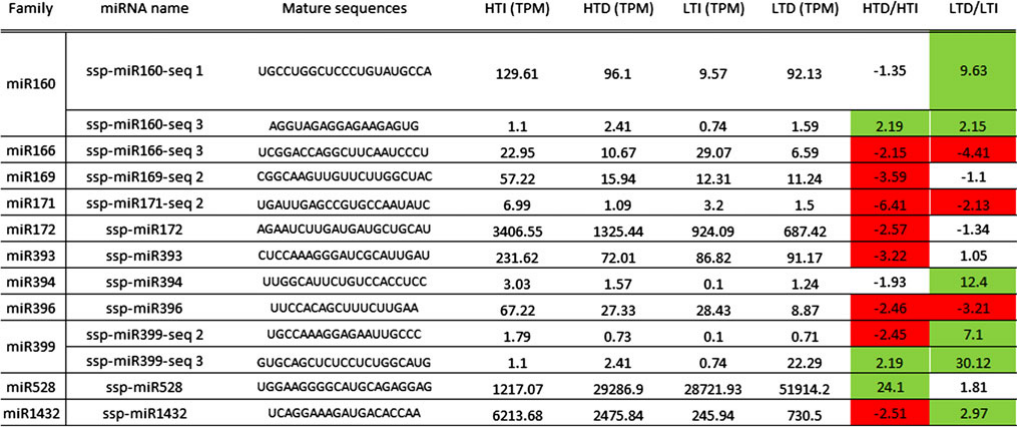
Differentially expressed mature microRNAs found under drought stress in sugarcane

HT cultivar RB867515, LT cultivar RB855536. Green boxes indicate upregulated under drought, red boxes indicate downregulated under drought, considering a *p* value <0.05 and fold change ≥2. Statistics were calculated between irrigated and drought treatments in each cultivar using the Audic–Claverie method

*HTI* irrigated higher tolerant, *HTD* drought higher tolerant, *LTI* irrigated lower tolerant, *LTD* drought lower tolerant, *TPM* transcripts per million

**Fig. 3:**
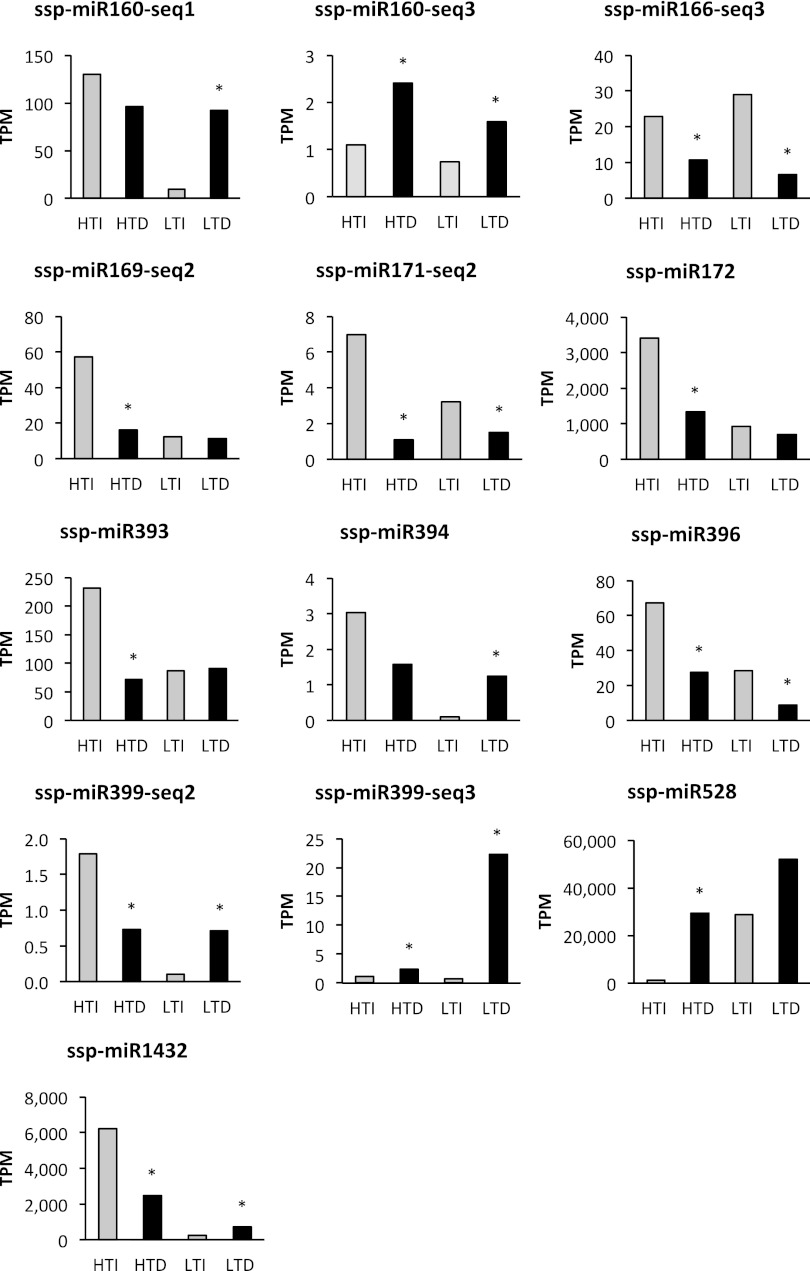
Expression profile based on the sequencing data of 13 differentially expressed sugarcane microRNAs. The value is expressed as the number of transcripts per million (TPM) for both conditions irrigated (control, *grey bars*) and drought-stressed (*black bars*) for the RB867515 (higher tolerance to drought) and RB855536 (lower tolerance to drought) cultivars. Each sample was a pool of two replicates. * *p* < 0.05, and fold change >2.0. Statistics were calculated between irrigated and drought treatments for each cultivar using the Audic–Claverie method

The sequencing results showed that ssp-miR160-seq 1, ssp-miR160-seq 3, ssp-miR394, ssp-miR399-seq 2, ssp-miR399-seq 3, ssp-miR528, and ssp-miR1432 were induced under drought stress compared to controls, in at least one cultivar (Fig. 3). The majority was induced only in the LT cultivar (RB855536), with the exception of ssp-miR528, which was expressed more highly under drought in the HT cultivar (RB867515). Only ssp-miR160-seq 3 and ssp-miR399-seq 3 were induced under drought in both cultivars.

In contrast, some miRNAs were repressed under drought treatment, and most of them were downregulated in the HT cultivar. Only ssp-miR166-seq 3, ssp-miR171-seq 2, and ssp-miR396 were repressed under drought in both cultivars (Fig. 3).

The expression profiles of two miRNAs (ssp-miR160-seq1 and ssp-miR528) modulated by drought were further analyzed by qRT-PCR. These miRNAs presented expression patterns that were similar to those observed with deep-sequencing (Supplementary Fig. S2).

### Prediction of sugarcane miRNA targets

Many of the differentially expressed miRNAs had target candidates in the SUCEST database, with the exception being ssp-miR160-seq 3, ssp-miR396 and ssp-miR399-seq 2 (Table 4). Among the targets, most of them encode transcription factors (ssp-miR160-seq 1, ssp-miR166-seq 3, ssp-miR169-seq 2, ssp-miR171-seq 2, ssp-miR172, ssp-miR528 and ssp-miR1432, among others). Other targets encode transporters (ssp-miR172, ssp-miR528, ssp-miR1432), proteins associated with senescence (ssp-miR399-seq 3) and proteins involved with flower development (ssp-miR172) (Table 4, Supplementary Table S2).

**Table 4:** Target prediction of the miRNAs differentially expressed under drought stress in field-grown sugarcane plants

miRNA name	Target Acce.	Expectation	UPE	Mature miRNA	Target start	Target end	miRNA aligned fragment	Target aligned fragment	Target description
ssp-miR160-seq 1	SCCCLRlC04H01.g	2.5	24.709	20	1,087	1,106	UGCCUGGCUCCCUGUAUGCC	GGCAGGCAGGCAGCCAGGCA	NAC domain-containing protein 68-like (*Brachypodium distachyon*), 68 %
ssp-miR166-seq 3	SCRFLR1034E12.g	3.0	22.151	20	875	894	UCGGACCAGGCUUCAAUCCC	UGGGAUGAAGCCUGGUCCGG	Homeobox-leucine zipper protein HOX32 (*Oryza sativa*), 99 %
ssp-miR169-seq 2	SCACST3157E03.g	2.5	16.856	21	121	141	CGGCAAGUUGUUCUUGGCUAC	GCAGCCAAGAAUGAUUUGCCU	Nuclear transcription factor Y subunit A-10 (*Zea mays*), 80 %
ssp-miR171-seq 2	SCJFAD1013C10.g	0.5	23.909	21	574	594	UGAUUGAGCCGUGCCAAUAUC	GAUAUUGGCGCGGCUCAAUCA	Scl1 protein (*Oryza sativa* Japonica Group), 74 %
ssp-miR172	SCJLRT1022F08.g	2.5	15.83	20	632	651	AGAAUCUUGAUGAUGCUGCA	UGCAGCAUCAUCACGAUUCC	Floral homeotic protein APETALA 2-like (*Brachypodium distachyon*), 67 %
ssp-miR393	TC120009	1.0	20.653	19	302	320	CUCCAAAGGGAUCGCAUUG	CAAUGCGAUCCCUUUGGAU	Auxin-responsive factor TIR1 protein (*Populustomentosa*), 84 %
ssp-miR394	SCUTLR1037A06.g	3.0	22.853	19	1,222	1,240	UU6GCAUUCUGUCCACCUC	GAGGUGGUCAGGAUGCUGG	Protein N5P-interacting kinase 1-like (*Brachypodium distachyon*), 87 %
spp-miR399-seq 3	SCJFLR1017A12.g	2.5	18.233	19	290	308	GUGCAGCUCUCCUCUGGCA	GGCCAGAAGGGAGCUGCAC	Senescence-associated-like protein (*Zea mays*), 95 %
ssp-miR528	SCCCCL1002D10.b	2.5	10.325	21	133	153	UGGAAGGGGCAU6CAGAGGAG	UUCCUCCGCACGCCCUUUCCA	Pyruvate dehydrogenase El alpha subunit (*Zea mays*), 99 %
ssp-miR1432	SCSFFL4085D03.g	3.0	15.088	19	624	642	UCAGGAAAGAUGACACCAA	UUGGUGUUUUCUUCCCUGA	bZIP transcription factorl (Zea mays), 85 %

Target Acc: accession number in the SUCEST or SoGI databases; Expectation: value assigned to the alignment of the mature miRNA and the target. The value ranges from 0 (perfect alignment) to 5, UPE: the energy needed to open the secondary structure of the target at the site recognition (less energy means better accessibility to the target); Mature miRNA: miRNA mature size (in nucleotides); Target start: the base position where the annealing with the miRNA starts; Target end: the base position where the annealing with the miRNA ends; Target description: description of the target according to a BLAST search in the GenBank database, including the name of the organism presenting the best hit

### Analysis of target gene expression

**Fig. 4:**
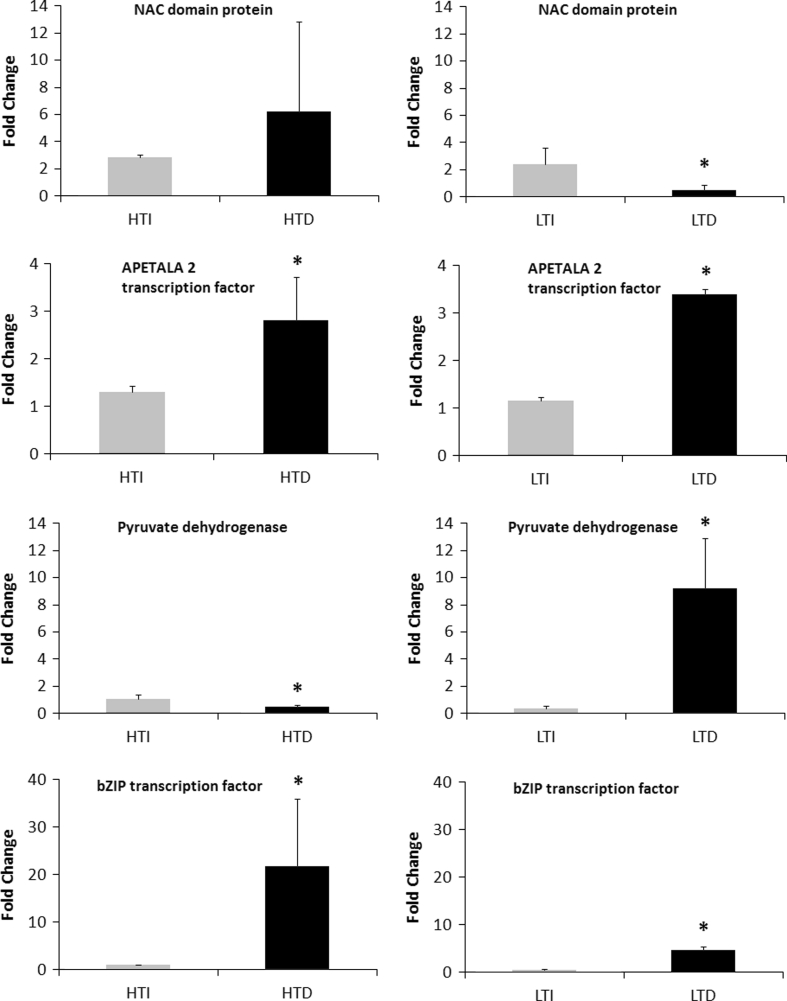
Expression profile of one of the predicted target genes for four sugarcane miRNAs modulated by drought. The values are expressed as fold changes relative to the irrigated control for each gene. The *bars* represent the average of the irrigated plants (control, *grey bars*) and drought-stressed plants (*black bars*) for RB867515 and RB855536 after 7 months of stress. *Error bars* represent the standard deviation (*n* = 3). Statistics were calculated between irrigated and drought-treated plants using a *t* test. *Asterisk* indicates the differences between irrigated and drought-stressed plants, with *p* ≤ 0.05

To evaluate whether the miRNA expression profiles correlated with differences in the transcripts from the target genes, the expression of four targets corresponding to the four miRNAs with the highest expression (miR160-seq 1, miR172, miR528, and miR1432; Fig. 3) were evaluated by RT-qPCR (Fig. 4). To facilitate the comparison between the expression of miRNAs and their corresponding target genes (Figs. 3, 4), we calculated the ratios between drought-stressed and irrigated expression levels (Table 5). Among the eight expression profiles, six cases behaved as expected, i.e., miRNA expression was induced and target gene expression was repressed upon drought stress and vice versa (shown in bold in Table 5). In four out of five cases where the miRNA profiles had a *p* value <0.05 (Fig. 3), the expected trend in the profile of the target gene was also significant at *p* < 0.05. In two cases, the miRNA and target gene profiles showed no agreement in the LT cultivar (Table 5), which suggests that the regulation by miRNA might be influenced by the genetic background.

**Table 5:** Expression profiles of selected target genes found for the miRNAs differentially expressed under drought conditions

miRNA target	SAS cluster name	Target description	HT7	LT7
Target miR160-seq 1	SCCCLRlC04H0l.g	NAC domain containing protein 68-like	**0.74/2.17**	9.62*/0.19*
Target miR172	SCJLRT1022F08.g	Floral homectic protein Apetala2	0.4*/2.16*	0.74/2.94*
Target miR528	SCCCCL1002D10.b	Pyruvate dehydrogenase E1 alpha subunit	24.06*/0.44*	1.80/23.5*
Target miR1432	SCSFFL4085D03.g	B-ZIP transcription factor	0.40*/20.1*	2.97*/7.86*

The SAS cluster name and the complete description of each target gene are described in the table. HT: RB867515 (higher drought tolerant plants); LT: RB855536 (lower drought tolerant plants). The expression ratios between drought-stressed and control plants are shown. The first number in each pair indicates miRNA levels and the second indicates the target gene expression. Asterisk indicates ratios where differences in the expression levels in the irrigated and drought-stressed plants are statistically significant (*p* < 0.05). The bold expression ratios indicate that miRNA induction or repression correlates with repression or induction of target genes, respectively. Statistics were calculated between irrigated and drought treatments using a *t* test

*SAS* sugarcane assembled sequence, *HT* higher tolerance cultivar, *LT* lower tolerance cultvar, 7, 7 months of stress

## Discussion

There are many recent reports confirming the identification of miRNAs in different plants, and some of these also describe differential expression patterns of miRNAs under stress (Jian et al. 2010; Jones-Rhoades and Bartel 2004; Matts et al. 2010; Pantaleo et al. 2010; Ruan et al. 2009; Unver and Budak 2009; Xin et al. 2010; Zhang et al. 2009b; Zhao et al. 2010; Zhou et al. 2010). Although a small number of sugarcane miRNAs have already been registered in miRBase, to the best of our knowledge, this is the first report that associates drought stress and miRNA identification in field-grown sugarcane cultivars based on sequencing profiles.

During the development of the Sugarcane Expressed Sequence Tag Project (SUCEST), 43,141 transcripts of sugarcane (SAS) were generated (Vettore et al. 2001). Approximately 30 % of the SAS had no significant identity to sequences from other organisms and might be involved in the post-transcriptional regulation of other genes. Currently, there are only 34 sugarcane miRNA sequences predicted by bioinformatics in the miRBase database, including *Saccharum officinarum* (sof-miR) and *Saccharum* spp. (ssp-miR). In this work, we have identified 30 mature miRNA sequences belonging to 18 sugarcane miRNA families expressed in leaves under drought stress (Table 2), with some precursors identified in SoGI and SUCEST databases. Because miRNA expression could be specific to tissue, age or development stage, it is expected that the number of sugarcane miRNAs would increase when other tissues were analyzed.

Several new miRNAs, not conserved among the species, have been identified along with their targets, which include genes associated with diverse metabolic pathways and cellular processes related with the development of resistance to abiotic stresses (Eckardt 2004; Llave et al. 2002; Sunkar and Zhu 2004). Using *P. trichocarpa* as a model, Lu et al. (2005) confirmed the in silico-predicted targets as genes related to development and/or stress, with putative associated functions as cell wall metabolites important in the regulation of wood development in trees. Although some miRNAs share conserved sequences, most of them exhibit species-specific expression profiles during development, suggesting that conserved miRNAs could have different regulatory roles in different species.

Sugarcane miRNA targets were predicted by psRNAtarget and some of these targets were validated by RT-qPCR (Table 5; Fig. 4). 10 out of 13 drought-modulated miRNAs predicted multiple target genes in the SUCEST database and 1 predicted target gene based on the SoGI database (Table 4, Supplementary Table S2). The majority of the targets encoded transcription factors, as already described for other organisms (Rhoades et al. 2002).

The expression profile of ssp-miR160-seq 1 was dependent on the cultivar and treatment, being significantly induced in the LT cultivar (RB855536) and slightly repressed in the HT cultivar (RB867515). One of the targets for this miRNA is a protein containing a NAC domain. Proteins from this family are known to be induced by diverse abiotic factors (Ditt et al. 2011; Hegedus et al. 2003; Sun et al. 2011). Although we found a high variability in the expression of the target gene in the field-grown plants, the ssp-miR160-seq 1 and target gene expression profiles indicated that LT plants repress and have lower levels of the target gene, while the opposite profile was observed in HT plants. Therefore, the gene encoding a NAC protein might play a role in the differences observed between the HT and LT cultivars.

ssp-miR166-seq 3 was repressed in both HT and LT cultivars and targets genes encoding homeobox-leucine zipper proteins, a family of transcription factors found only in plants. Transgenic Arabidopsis plants overexpressing the *Helianthus annus* *Hahb*-*4* gene presented increased tolerance to water stress (Dezar et al. 2005). The downregulation of ssp-miR166-seq 3 in both cultivars, leading to higher levels of the homeobox-leucine zipper protein, would enhance sugarcane tolerance to drought stress.

Previous studies in rice (*O. sativa*) showed that some members of the miR169 family were induced by drought (Zhao et al. 2007a) and salt stress (Zhao et al. 2009), while other members of this family were repressed (Li et al. 2008; Zhao et al. 2007a). ssp-miR169-seq 2 target genes belong to the Nuclear Factor YA family (NF-Y), a group of transcription factors that have three distinct subunits (NF-YA, NF-YB, and NF-YC) that bind to the CCAAT box (Combier et al. 2006; Li et al. 2008). Nine *NF*-*Y* genes expressed in wheat leaves responded to drought stress (Stephenson et al. 2007). The *NF*-*YA* genes have been reported to be involved in plant drought resistance, and the overexpression of NF-YA5 and NF-YB1 in *Arabidopsis* can also provide drought tolerance (Li et al. 2008; Nelson et al. 2007). Another interesting putative target encodes a glutathione S-transferase. These enzymes are well known for their role in protecting plants from oxidative stress. In sugarcane, we found that ssp-miR169 was downregulated in the HT cultivar (RB867515) during water deficit (Fig. 3; Table 3), suggesting that its target genes were upregulated. This upregulation suggests increased expression of *NF*-*Y* genes and lower oxidative stress in the HT plants, implicating these genes in sugarcane drought tolerance. However, ssp-miR169-seq 2 expression in LT plants was unchanged. Moreover, ssp-mR169-seq 2 levels were similar to what was observed in HT plants under drought stress, indicating that the target genes would be expressed at similar levels in both sugarcane cultivars during drought stress.

ssp-miR171-seq 2 was downregulated after 7 months of stress in both cultivars. A target gene for this miRNA encodes a protein with similarity to members of the GRAS/SCL family (Table 4). Members of this family include transcription factors that participate in diverse plant growth pathways and respond to stress, namely abiotic stress. The Scarecrow-like genes (*SCL*) belong to the GRAS multigenic family that are named for the following three loci: Gibberellic-acid insensitive (GAI), the GAI repressor (RGA) and the Scarecrow (SCR) (Pysh et al. 1999). Two of the members of the GRAS family, GAI and RGA, participate in the Gibberellic acid (GA) signal transduction pathway. The rice SLR1 was identified as GAI orthologue and is involved in the GA pathway in maize, grape, wheat, and barley (Hynes et al. 2003). In poplars, the *PeSCL7* gene was induced by drought and salt stress, and conferred tolerance to these stressors when overexpressed in Arabidopsis (Ma et al. 2010). The downregulation of ssp-miR171-seq 2, by increasing the levels of the sugarcane *SCL* target gene, which may activate other genes, might contribute to an increase in drought tolerance in both cultivars.

We found that ssp-miR172 was downregulated in both cultivars under drought conditions, with a major change in the HT cultivar (RB867515) (Fig. 3; Table 3). There are three transcription factors among the predicted target genes for this particular miRNA. One of them belongs to the APETALA2 family of transcription factors (Table 4) and was induced in both cultivars (Fig. 4; Table 5). In rice, osa-miR172 was downregulated by water deficit stress (Zhou et al. 2010). The APETALA2 family is one of the largest families of transcription factors in Arabidopsis, with 145 loci (Sakuma et al. 2002). Some members, such as the *DREB* genes, are involved in plant responses to drought and salt stress (Krishnaswamy et al. 2011). Therefore, ssp-miR172 may increase the expression of transcription factors that activate plant responses to drought stress.

ssp-miR393 presented no changes in the LT cultivar and was significantly repressed in the HT cultivar under drought (Fig. 3). One of the putative targets encodes a protein similar to TIR1, an auxin receptor in *A. thaliana* (Table 4). TIR1 recognizes 3-indole-acetic acid (AIA) and promotes the degradation of the Aux/AIA repressor by a protein ubiquitin ligase that binds to a conserved area of the repressor and allows transcription of auxin-regulated genes (Dharmasiri et al. 2005). Recently, the expression of *TIR1* was associated with the response of *A. thaliana* roots to inorganic phosphate (P*i*) (Perez-Torres et al. 2008). Our data suggest that in HT cultivars, ssp-miR393 is involved in the regulation of genes that modulate auxin activity in leaves during drought stress.

ssp-miR394 was downregulated under drought stress in the HT sugarcane cultivar and significantly upregulated in the LT cultivar, showing a different response between these genotypes. Among the predicted targets of ssp-miR394 is a gene encoding the protein NSP-interacting kinase (NIK) (Table 4). NIK belongs to a receptor-like serine/threonine kinase subfamily, the members of which contain five leucine-rich repeats that are involved in plant development and the response to biotic stresses, namely viral stress (Santos et al. 2010). This is the first study relating this kinase with drought response.

ssp-miR399-seq 3 was induced in both cultivars, and had significantly higher levels in the LT cultivar. A putative target of ssp-miR-399-seq 3 encodes a senescence-associated protein (Table 4). Leaf senescence is a symptom of water deficit and transgenic tobacco plants with delayed leaf senescence had increased tolerance to drought (Rivero et al. 2010). The upregulation of ssp-miR399-seq 3, by decreasing the levels of the senescence-associated protein, might contribute to an increase in drought tolerance in sugarcane.

ssp-miR528 has several putative targets, and we have evaluated by RT-qPCR the gene encoding a pyruvate dehydrogenase. This enzyme is part of a pyruvate dehydrogenase complex (PDHc) that plays a pivotal role in cell metabolism, catalyzing the oxidative decarboxylation of pyruvate and the subsequent acetylation of coenzyme A to acetyl-CoA (Gutowski and Lienhard 1976; Nemeria et al. 2001). Pyruvate dehydrogenase links the glycolysis metabolic pathway to the citric acid cycle, where the acetyl-CoA is used to carry out cellular respiration. In this work, both sugarcane cultivars showed an increase in ssp-miR528 expression under drought conditions, where the HT cultivar has a higher increase than the LT cultivar. This expression increase may prevent loss of CO_2_ to the atmosphere by leaf respiration, leading to better control of carbon balance during drought stress (Chaves et al. 2002, 2009; Flexas et al. 2006).

The ssp-miR1432 miRNA was downregulated under drought stress in HT plants and induced in the LT cultivar. This miRNA also has several putative targets, and we have evaluated one target encoding a bZIP transcription factor that has been shown to confer stress tolerance to plants (Golldack et al. 2011). The ssp-miR1432 expression pattern indicates that HT plants adjust their transcriptome to increase the bZIP factor, which may activate the transcription of drought-related genes.

Recently, we have evaluated sugarcane miRNAs that had their expression patterns modulated by drought in 3-month-old plants grown in a greenhouse (Ferreira et al. 2012). Seven drought-responsive miRNAs were identified in these plants: ssp-miR164, ssp-miR393, ssp-miR394, ssp-miR397, ssp-miR399-seq 1, ssp-miR528, and ssp-miR1432. Interestingly, four miRNAs (ssp-miR393, ssp-miR394, ssp-miR528, and spp-miR1432) were modulated by drought in both greenhouse and field experiments, but only ssp-miR528 (up-regulated) and ssp-miR1432 (down-regulated) had similar expression patterns in both experiments, and only in the HT cultivar. In another work using sugarcane 3-month-old plants, roots exposed to drought stress for 24 h presented five miRNAs that were differentially expressed (Thiebaut et al. 2012). None of them were observed in the leaves from plants grown under field conditions. These findings indicate that sugarcane response to drought is affected by plant age and organ, and also by the experimental conditions.

In summary, we have studied two cultivars grown in the field for 7 months that differ in drought tolerance, with or without irrigation. Field experiments provide more reliable information on the effect of stress on plants in a natural environment. We have shown that the expression patterns of several miRNAs are modulated by drought in the field and that some may play a significant role in the higher drought tolerance observed in the RB867515 cultivar. Finally, we showed evidence that miRNA expression profiles may vary according to the genetic background from the distinct sugarcane cultivars.

## Electronic supplementary material

Below is the link to the electronic supplementary material.

Supplementary material 1 (DOCX 15 kb)

Supplementary Fig. S1 Physiological data analyzed in field-grown sugarcane cultivars RB867515 (higher tolerance to drought, HT) and RB855536 (lower tolerance to drought, LT) under irrigated or water-deficit conditions for 7 months. *Error bars* represent the standard error (*n* = 4). *Asterisk* indicates significant differences calculated by a *t* test between irrigated and drought-stressed plants where *p* < 0.05. Monthly values of rainfall and reference evapotranspiration (ETo) were estimated by a Class A evaporation pan. Rain fall—ETo refers to the deficit of water in the ambient at Campo Alegre, Alagoas, Brazil (TIFF 159 kb)

Supplementary Fig. S2 RT-qPCR expression profiles of two sugarcane miRNAs modulated by drought stress. The values are expressed as fold changes relative to the irrigated control for each gene. The *bars* represent the average of the irrigated plants (control, *grey bars*) and drought-stressed plants (*black bars*) for RB867515 (TH, higher drought tolerance) and for the RB855536 (LT, lower drought tolerance) after 7 months of stress. *Error bars* represent the standard error (*n* = 3). Statistics was calculated between irrigated and drought treatments in each cultivar using the *t* test (TIFF 39 kb)

Supplementary Table S1 RT-qPCR primers used in sugarcane genes expression analysis. *RT* primer loop for reverse transcription, *FW* forward primer for real-time PCR, *RV* reverse primer for real-time PCR, *PUB* polyubiquitin gene, *tgt* target gene. The complete sequence, the number of nucleotides (Nt) and the melting temperature (Tm) of each primer are shown (TIFF 53 kb)

Supplementary Table S2 Target prediction of the miRNAs differentially expressed after drought stress in field-grown sugarcane plants. All the data obtained from the bioinformatics analysis without selection are presented here. Target Acc: accession number in the SUCEST or SoGI databases; Expectation: value assigned to the alignment of the mature miRNA and the target where the value ranges from 0 (perfect alignment) to 5; UPE: the energy needed to open the secondary structure of the target at the site recognition (less energy indicates better accessibility to the target); Mature miRNA: miRNA mature size (in nucleotides); Target start: the position where annealing with the miRNA starts; Target end: the position where annealing with the miRNA ends; Target description: description of the target according to a BLAST search in the GenBank database, including the name of the organism presenting the best hit (TIFF 483 kb)
