# Supplementary material for: Effects of drought on the microtranscriptome of field-grown sugarcane plants
Source: Planta. 2012 Nov 6;237(3):783–98. doi: 10.1007/s00425-012-1795-7 (PMC3579473; doi:10.1007/s00425-012-1795-7)
Supplement: Supplementary file 1 — Supplementary material 1 (DOCX 15 kb) [file 425_2012_1795_MOESM1_ESM.docx]

Supplementary Material

Supplementary Fig S1. Physiological data

The experiment was conducted at the Federal University of Alagoas (UFAL) under field-grown conditions with the RB867515 cultivar (HT, higher tolerance to drought) and the RB855536 cultivar (LT, lower tolerance to drought) with or without irrigation. Seven months after planting, IRGA (LCpro+, ADC Bioscientific, UK) was used to obtain the photosynthetic rate (A), stomatal conductance (gs) and transpiration rate (E) data in leaf +1. Water (ψ_w_) and osmotic potential (ψ_os_) were also determined in leaf +1. The potentials were measured in early morning (4-5 h) and noon (12-13 h), with a Scholander Pump and a pressure osmometer (Vapro 5520 Wescor), respectively. The amount of proline was also determined.

In general, a water deficit reduced photosynthesis, stomatal conductance and transpiration rate in the mature leaves of seven-month-old field-grown plants (Supplementary Fig. S1, a-c). When submitted to drought, the cultivars showed a large decrease in the stomatal conductance. This stomatal closure is a conservation strategy that functions to reduce transpiration, increasing the water availability in the plant.

Drought reduced the photosynthetic rate (A) by nearly 50% in plants stressed for seven months (Supplementary Fig. S1, a). The LT cultivar (RB855536) showed even lower rates of photosynthesis. This demonstrates that one of the first responses of these sugarcane cultivars might be stomatal closure, which minimizes the loss of water and reduces photosynthesis. The transpiration rate (E) was also reduced under drought stress in both cultivars (Supplementary Fig. S1, c), although the HT cultivar (RB867515) presented a higher transpiration rate compared with the LT cultivar (RB855536).

We found differences in the water potential (ψ_w_) at seven months between treatments (Fig. 1S, e,f). The cultivars behaved differently between both measurements (early morning and noon). The water potential measured early in the morning showed no significant differences between treatments in either cultivars (Fig. 1S, e). This response could be due to the soil conditions and/or a deficiency in the irrigation system, considering that the early water potential should be equivalent to the soil potential after equilibrium was achieved during the night. At noon, when the plant is submitted to intense sun radiation and under water deficiency conditions, we observed a significant reduction in the water potential under drought treatment (Supplementary Fig. S1, f). However, in the HT cultivar, the differences were not significant between treatments, indicating that under these stress conditions, plants could adjust osmotically and increase the gradient between plant and soil, resulting in better water absorption (Fig. S1, f).

Under water deficiency conditions, plants presented a higher solute concentration in the leaves (more negative values) at both time points (5 and 12 h) (Supplementary Fig. S1, g,h). The osmotic potential was reduced in the early morning (5 h) and at noon (12 h) under drought stress in both cultivars. The LT cultivar (RB855536) presented more negative osmotic potentials (ψ_os_), likely indicating a greater damage due to drought stress. When submitted to long periods of water deficiency, plants had a reduction in osmotic potential in an attempt to reduce the leaf water potential, which may be one of the drought-stress tolerance mechanisms in sugarcane.

Plants stressed with drought conditions after seven months had higher concentrations of proline (Supplementary Fig. S1, d), especially in the LT cultivar (RB855536), in which the differences between treatments were significant.

These negative effects observed in the rainfed plants reflect the water stress that these plants were subject over the growing period. As shown in Fig. S1 (i), except for February/2009, during all months the evapotranspiration from sugarcane plants were higher than rainfall levels. This stressful condition reduced the crop productivity in both cultivars, although the HT cultivar had higher productivity than the LT cultivar, as expected (Fig. S1, j).

In summary, water deficiency negatively affected the photosynthetic activity. Drought conditions reduced the gs, A, E, water and osmotic potentials, indicating a reduction in the photosynthetic performance. Under these conditions, plants implemented a conservation mechanism, reducing the stomatal conductance and transpiration and increasing the efficiency in water consumption.
