# Supplementary figures and images for: Effects of drought on the microtranscriptome of field-grown sugarcane plants
Source: Planta. 2012 Nov 6;237(3):783–98. doi: 10.1007/s00425-012-1795-7 (PMC3579473; doi:10.1007/s00425-012-1795-7)

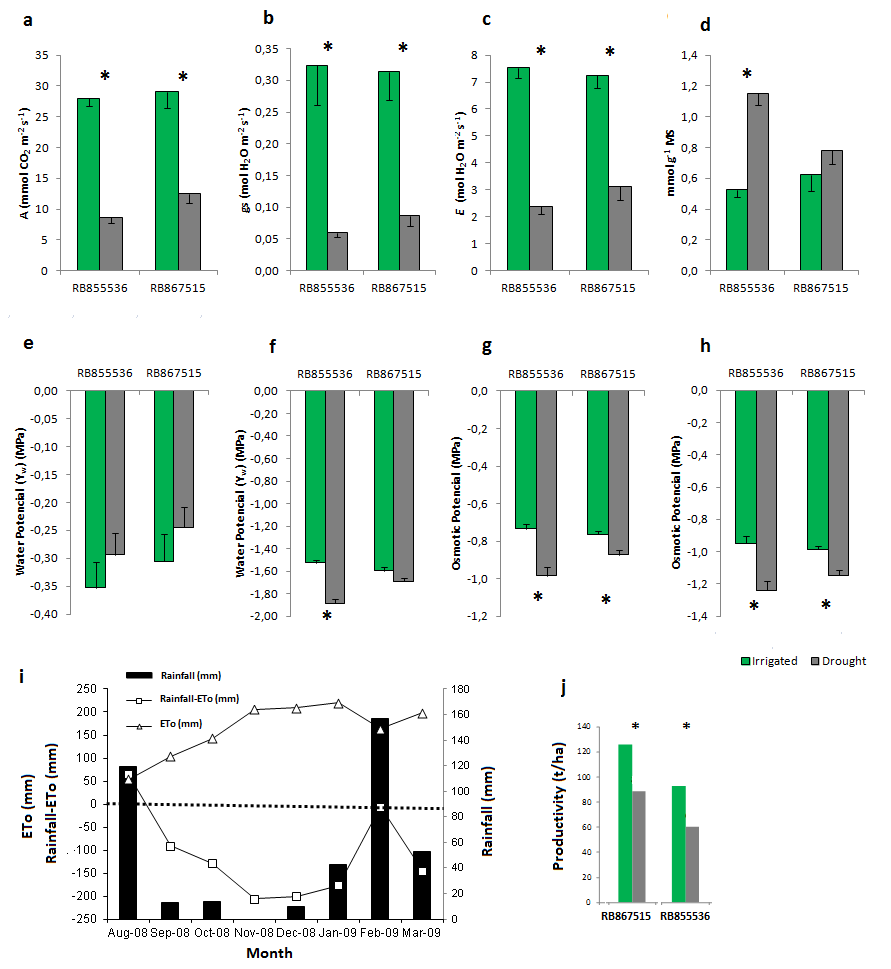

Supplement: Supplementary file 2 — Supplementary Fig. S1 Physiological data analyzed in field-grown sugarcane cultivars RB867515 (higher tolerance to drought, HT) and RB855536 (lower tolerance to drought, LT) under irrigated or water-deficit conditions for 7 months. Error bars represent the standard error (n = 4). Asterisk indicates significant differences calculated by a t test between irrigated and drought-stressed plants where p < 0.05. Monthly values of rainfall and reference evapotranspiration (ETo) were estimated by a Class A evaporation pan. Rain fall—ETo refers to the deficit of water in the ambient at Campo Alegre, Alagoas, Brazil (TIFF 159 kb) [file 425_2012_1795_MOESM2_ESM.tif]

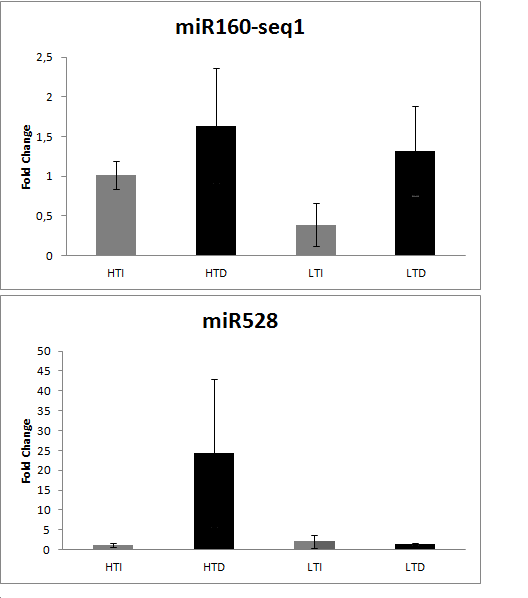

Supplement: Supplementary file 3 — Supplementary Fig. S2 RT-qPCR expression profiles of two sugarcane miRNAs modulated by drought stress. The values are expressed as fold changes relative to the irrigated control for each gene. The bars represent the average of the irrigated plants (control, grey bars) and drought-stressed plants (black bars) for RB867515 (TH, higher drought tolerance) and for the RB855536 (LT, lower drought tolerance) after 7 months of stress. Error bars represent the standard error (n = 3). Statistics was calculated between irrigated and drought treatments in each cultivar using the t test (TIFF 39 kb) [file 425_2012_1795_MOESM3_ESM.tif]

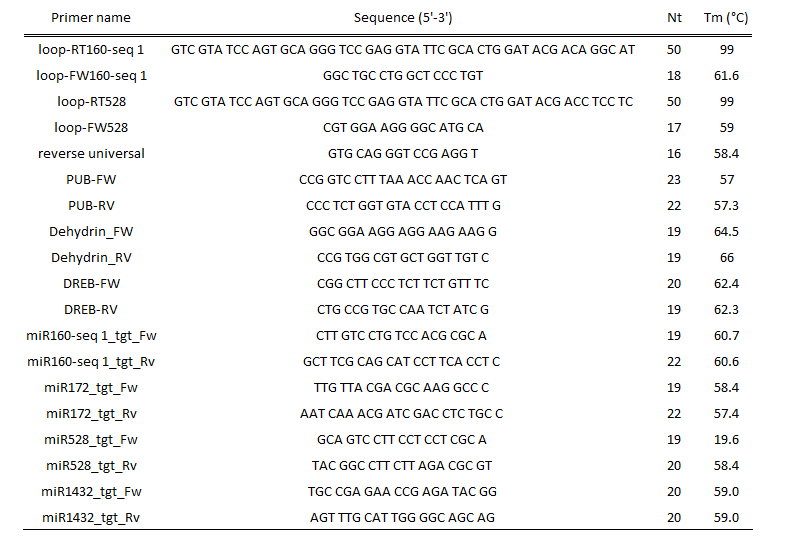

Supplement: Supplementary file 4 — Supplementary Table S1 RT-qPCR primers used in sugarcane genes expression analysis. RT primer loop for reverse transcription, FW forward primer for real-time PCR, RV reverse primer for real-time PCR, PUB polyubiquitin gene, tgt target gene. The complete sequence, the number of nucleotides (Nt) and the melting temperature (Tm) of each primer are shown (TIFF 53 kb) [file 425_2012_1795_MOESM4_ESM.tif]

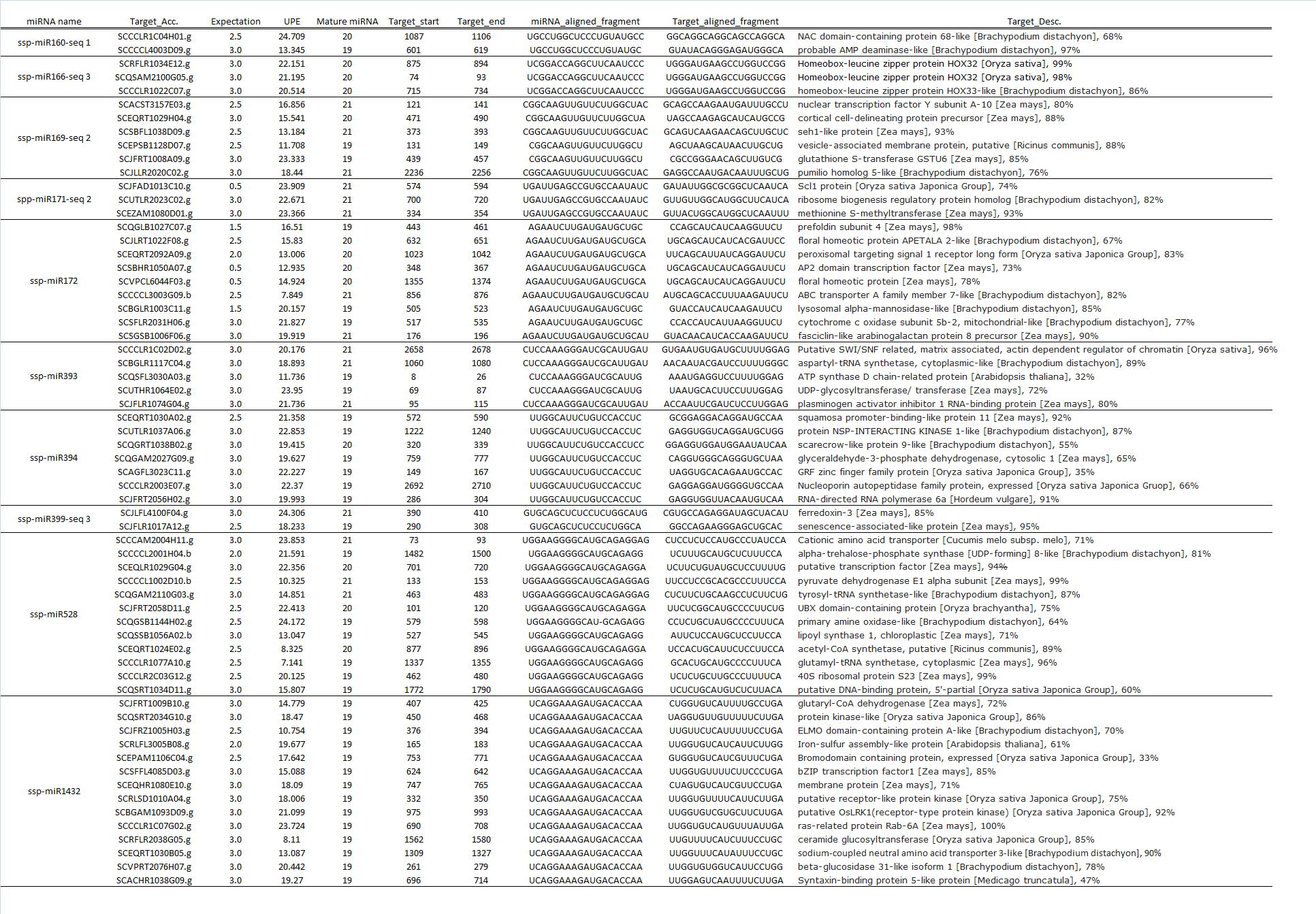

Supplement: Supplementary file 5 — Supplementary Table S2 Target prediction of the miRNAs differentially expressed after drought stress in field-grown sugarcane plants. All the data obtained from the bioinformatics analysis without selection are presented here. Target Acc: accession number in the SUCEST or SoGI databases; Expectation: value assigned to the alignment of the mature miRNA and the target where the value ranges from 0 (perfect alignment) to 5; UPE: the energy needed to open the secondary structure of the target at the site recognition (less energy indicates better accessibility to the target); Mature miRNA: miRNA mature size (in nucleotides); Target start: the position where annealing with the miRNA starts; Target end: the position where annealing with the miRNA ends; Target description: description of the target according to a BLAST search in the GenBank database, including the name of the organism presenting the best hit (TIFF 483 kb) [file 425_2012_1795_MOESM5_ESM.tif]
